# The Role of Hypoxia Inducible Factor-1 in Hepatocellular Carcinoma

**DOI:** 10.1155/2014/409272

**Published:** 2014-07-02

**Authors:** Dongjun Luo, Zhongxia Wang, Junyi Wu, Chunping Jiang, Junhua Wu

**Affiliations:** ^1^Department of Hepatobiliary Surgery, Nanjing Drum Tower Hospital Clinical College of Nanjing Medical University, Nanjing, Jiangsu 210008, China; ^2^Department of Hepatobiliary Surgery, The Affiliated Drum Tower Hospital of Nanjing University Medical School, Nanjing, Jiangsu 210008, China; ^3^Medical School, Nanjing University, Nanjing, Jiangsu 210093, China

## Abstract

Hypoxia is a common feature of many solid tumors, including hepatocellular carcinoma (HCC). Hypoxia can promote tumor progression and induce radiation and chemotherapy resistance. As one of the major mediators of hypoxic response, hypoxia inducible factor-1 (HIF-1) has been shown to activate hypoxia-responsive genes, which are involved in multiple aspects of tumorigenesis and cancer progression, including proliferation, metabolism, angiogenesis, invasion, metastasis and therapy resistance. It has been demonstrated that a high level of HIF-1 in the HCC microenvironment leads to enhanced proliferation and survival of HCC cells. Accordingly, overexpression, of HIF-1 is associated with poor prognosis in HCC. In this review, we described the mechanism by which HIF-1 is regulated and how HIF-1 mediates the biological effects of hypoxia in tissues. We also summarized the latest findings concerning the role of HIF-1 in the development of HCC, which could shed light on new therapeutic approaches for the treatment of HCC.

## 1. Introduction

Hepatocellular carcinoma (HCC) is one of the most aggressive malignancies and the third leading cause of cancer-related death worldwide [1]. In recent decades, HCC is characterized by poor prognosis and recurrence after liver resection. With advances in the development of molecular-targeted drugs, a lot of new diagnostic and therapeutic molecular targets may provide novel therapies in HCC [2].

**Figure 1:**
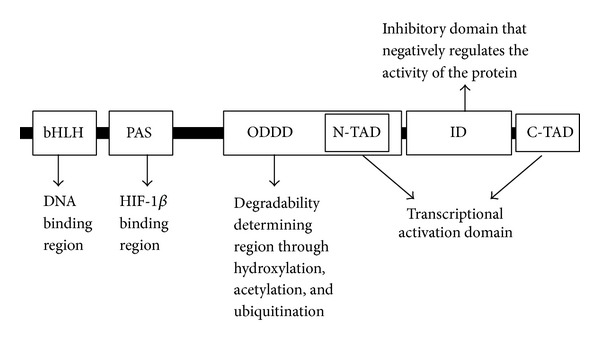
Domain structures of HIF-1*α* and their potential function in stability and transcriptional activity of HIF-1.

HCC shares the character of tissue hypoxia with other solid tumors, especially when the tumor grows quickly and angiogenesis fails to catch up with the speed of tumor growth. On one hand, hypoxia induces tumor necrosis which limits the size of HCC. However, on the other hand, tough hypoxic environment leads HCC cells to turn on hypoxia response, which subsequently leads to prosurvival reactions, elevated angiogenesis, adapted metabolic alteration, tumor invasion, and metastasis [3]. Hypoxia inducible factor-1 (HIF-1) is the first identified mediator of cell response to hypoxia in mammalian cells cultured under reduced oxygen tension [4]. This transcription factor is a heterodimer composed of two subunits: an oxygen-sensitive HIF-1*α* and a constitutively expressed HIF-1*β* which is also called aryl hydrocarbon receptor nuclear translocator (ARNT). Both HIF-1 subunits contain the basic helix-loop-helix (bHLH) and PER-ARNT-SIM (PAS) domains that are required for dimerization and combination with their corresponding DNA sequences, namely, hypoxia response element (HRE) in the promoter region of target genes [5]. HIF-1*α* has two independent transactivation domains located in its COOH-terminal portion: the NH2-terminal transactivation domain (N-TAD) and the COOH-terminal transactivation domain (C-TAD) [6]. The N-TAD constitutes the degradation box and is involved in the stabilization of HIF-1*α*, and the C-TAD functions in modulating the transcriptional activation of HIF-1*α* under hypoxic conditions. Under normoxia, the inhibitory domain (ID) located between the two TADs negatively regulates the activity of the protein. The oxygen-dependent degradation domain (ODDD) is required for degradation by the ubiquitin-proteasome pathway under normoxic conditions (Figure 1).

This review focuses on the activities that HIF-1 exerts in HCC, paying close attention to small-molecule inhibitors of the HIF-1 pathway as well as the recent progress of gene therapy aimed directly at HIF-1 genes.

## 2. Regulation and Target Genes of HIF-1 

### 2.1. The Degradation and Stability of HIF-1

**Figure 2:**
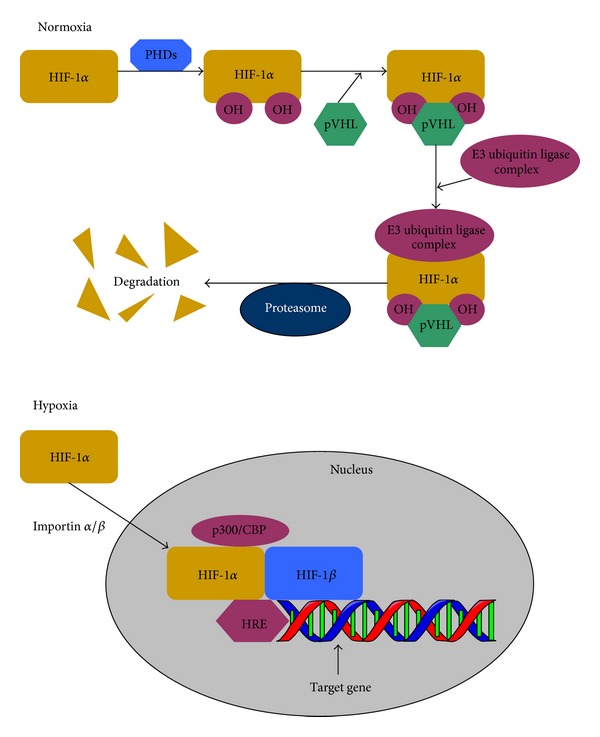
Oxygen-dependent regulation of HIF-1*α* activity. Under normoxic conditions, HIF-1*α* subunit is rapidly hydroxylated by prolyl hydroxylases (PHDs) and binds to von Hippel-Lindau protein (pVHL), resulting in the rapid ubiquitination of HIF-1*α* and subsequent proteasomeal degradation. Under hypoxic conditions, HIF-1*α* is stabilized and translocated into the nucleus by importin *α*/*β* and dimerizes with HIF-1*β*. The HIF heterodimer affects transcription of target genes by binding to a hypoxia response element (HRE) in the upstream promoter region after cooperation with transcriptional coactivators such as p300/CBP.

Under normoxic conditions, posttranslational HIF-1*α* is rapidly degraded by the proteasome and usually not detectable. In the absence of other metabolic perturbations, proline residues 402 and 564 at the ODDD of HIF-1*α* are hydroxylated by prolyl hydroxylases (PHDs). Hydroxylation of these two prolyl residues mediates binding of von Hippel-Lindau tumour suppressor protein (pVHL), which is the recognition component of the E3 ubiquitin ligase complex that targets HIF-1*α* for ubiquitination and degradation by the 26S proteasome [7] (Figure 2). In this process of degradation, three PHDs (PHD1, PHD2, and PHD3) are considered as the oxygen sensors regulating HIF-1*α*. These three homologs are identified recently in mammals and have the potential to hydroxylate HIF-1*α*. Among them, PHD2 has been shown to be the key limiting enzyme setting the low steady-state levels of HIF-1*α* in normoxia [8]. The activity of PHD2 is governed by the oxygen concentration within the cell, and the reaction converting proline into hydroxyproline also requires iron, 2-oxoglutarate, and ascorbate. Under hypoxic conditions, the inactivation of PHDs frees HIF-1*α* form hydroxylation, preventing pVHL binding to prolyl residues, which leads to HIF-1*α* stabilization in the cytoplasm. Based on the oxygen-dependent pVHL pathway, ARD1 (arrest defective 1) has been shown as a HIF-1*α* acetyltransferase stimulating HIF-1*α* degradation through the acetylation of Lys532, which enhances interaction of HIF-1*α* with pVHL [9].

In addition to the pVHL pathway, the primary mechanism by which HIF-1*α* keeps stable, some signaling proteins such as small ubiquitin-like modifier-1 (SUMO-1) and RWD-containing sumoylation enhancer (RSUME) also can control levels of HIF-1*α*. The ectopic expression of SUMO-1 increases HIF-1*α* stability and enhances its transcriptional activity by the cotransfection study, suggesting that sumoylation at Lys(391) and Lys(477) residues in the ODDD modulates other posttranslational modifications of HIF-1*α* [10]. RSUME is induced under hypoxia and promotes the sumoylation of HIF-1*α*, leading to its enhanced stabilization and transcriptional activity [11]. On the contrary, new evidence demonstrates that SUMO-specific protease 1 is essential for stabilization of HIF-1*α* during hypoxia, indicating that sumoylation can also target HIF-1*α* for ubiquitination and degradation [12].

Another pVHL-independent pathway for degradation of HIF-1*α* involves receptor of activated protein kinase C (RACK1) and heat shock protein 90 (Hsp90). As a HIF-1*α*-interacting protein, RACK1 promotes the ubiquitination and degradation of HIF-1*α* through recruitment of the Elongin-C/B ubiquitin ligase complex, whereas Hsp90 upregulates HIF-1*α* protein stability through competition with RACK1 for binding to the PAS domain of HIF-1*α*, which predicts that Hsp90 antagonists may promote loss of HIF-1*α* protein [13, 14].

### 2.2. The Transcriptional Activity of HIF-1

Hypoxia allows HIF-1*α* to escape recognition by the E3 ubiquitin ligase complex and translocate from cytoplasm to the nucleus. Nucleocytoplasmic shuttling of HIF-1*α* regulates its transcriptional activity by the classical nuclear transport receptors importin *α*/*β*, which directly interact with HIF-1*α* dependent on a functional nuclear localization signal (NLS) in the C-terminal region of HIF-1*α*. In contrast, the supposed NLS in the N-terminal region is not effective [15].

Under the effect of the mitogen-activated protein kinase (MAPK) pathway, HIF-1*α* would be directly phosphorylated, so that it can accumulate in the nucleus and is free to form heterodimer with HIF-1*β*. Subsequently, the C-TAD of HIF-1*α* is able to interact with transcriptional coactivators such as p300/CBP, resulting in increased HIF-1 transactivation after binding to HREs on the target genes (Figure 2). But under normoxic conditions, this interaction cannot occur in the nucleus due to the hydroxylation of the asparagines-803 residue at the C-terminal TAD by an asparaginyl hydroxylase termed factor inhibiting HIF-1 (FIH-1). FIH-1 contains certain motifs present in iron and 2-oxoglutarate-dependent oxygenases and blocks the binding of p300/CBP to HIF-1, leading to the cease of HIF-1-mediated gene transcription. However, under hypoxic conditions, the activity of FIH-1 is directly limited, allowing the interaction between HIF-1 and p300/CBP [16]. Since the activity of HIF-1 is regulated by two oxygen-dependent hydroxylation events, the PHDs have been shown to be more effective as oxygen sensors than the FIH-1 according to the differences between FIH and PHDs in their Michaelis constant values for oxygen and inhibition by 2-oxoglutarate analogs [17]. Furthermore, the in vitro study has shown that prolyl hydroxylation is substantially more sensitive than asparaginyl hydroxylation in the order of Pro (402) > Pro (564) > Asn (803) at the three sites in HIF-1*α* proteins [18].

### 2.3. The Target Genes of HIF-1

The HIF-1 complex acts as a transcription factor for many target genes in several aspects of cancer progression including angiogenesis, erythropoiesis, glucose metabolism, cell proliferation, and apoptosis [19, 20] (Table 1). Among these genes, vascular endothelial growth factor (VEGF) is one of the major target genes for HIF-1 that directly participates in angiogenesis [21]. However, HIF-1 contributes to angiogenesis by more complex mechanisms through the production of nitric oxide synthase (NOS), endothelin-1 (ET-1), stromal cell-derived factor-1 (SDF-1), angiopoietin 2 (ANGPT2), platelet derived growth factor (PDGF), leptin, and so forth than simple VEGF induction [22–27]. In addition to the activation of gene expression for angiogenesis, another essential process of cancer biology that HIF-1 activates is glucose metabolism. HIF-1 regulates the expression of many enzymes in the glycolytic pathway, which allows tumors to survive under hypoxia by metabolizing glucose to lactate through anaerobic glycolysis [28–30]. HIF-1 activates the glucose transporter 1 (GLUT1), which mediates cellular glucose uptake, as well as hexokinase (HK) and lactate dehydrogenase A (LDHA), which convert pyruvate to lactate. Regulated by HIF-1, the lactate can be removed from the cell through the action of monocarboxylate transporter 4 (MCT4) while pyruvate dehydrogenase kinase 1 (PDK1) and MAX interactor 1 (MXI1) can block the flow of pyruvate into the mitochondria [31–36]. Several hypoxia-induced growth factors, most notably insulin-like growth factor-2 (IGF-2), transforming growth factor (TGF), C-MYC, and inhibitor of differentiation 2 (ID2), which are known to promote cell proliferation, are also HIF-1 target genes [37–40]. In contrast, HIF-1 also leads to cell growth arrest and apoptosis through upregulating the expression of several target genes such as p53, BNIP3, and Caspase 3 [41–43]. Furthermore, hypoxia unleashes the invasive and metastatic potential of tumor cells. HIF-1 regulates the expression of genes encoding matrix metalloproteinase 2 (MMP2), fibronectin 1 (FN1), C-MET, autocrine motility factor (AMF), and keratins 14 (KRT14), which play established roles in the pathophysiology of invasion [44–48].

## 3. Role of HIF-1 in HCC

### 3.1. HIF-1 and Cellular Proliferation and Apoptosis

Compelling evidence indicates that HIF-1 expression is intimately correlated with proliferation and apoptosis of cancer cells. However, the role of HIF-1 on cellular proliferation and apoptosis of hepatoma cells remains controversial. In most cases, HIF-1 acts as an antiapoptotic factor. In the work by Xia and coworkers, it is illustrated that Forkhead box M1 (FoxM1) acts as a proliferation-specific transcription factor in cell growth of HCC and HIF-1 directly binds to the FoxM1 promoter induced by tumor necrosis factor-*α* (TNF-*α*), which suggests that the TNF-*α*/HIF-1-induced upregulation of FoxM1 expression may promote the proliferation of hepatoma cells and their resistance to apoptosis [49]. By using a Tet-on inducible system to regulate HIF-1*α* expression in the HepG2 cells in vitro under hypoxia; Xu and coworkers showed that HIF-1 promotes cell proliferation and accelerates the cell cycle through influencing the expression of cyclin A and cyclin D. Moreover, the assay of caspase activity indicates that HIF-1 suppresses hepatocellular cell apoptosis by upregulating survivin and Bcl-2 expression [50]. Several studies have demonstrated that primary hepatocellular carcinoma needs Omi/HtrA2 expression for cell apoptosis and HIF-1 inhibits the apoptotic process in HCC cells through upregulating Bcl-2 expression to impede Omi/HtrA2 releasing from the mitochondrion [51, 52]. In another work by Jeon and coworkers, the role of HIF-1 is demonstrated in the mechanism of sulforaphane (SFN)-induced apoptosis in human hepatoma cells. SFN downregulates the expression of HIF-1*α*, which may strongly inhibit 6-phosphofructo-2-kinase/fructose-2, 6-bisphosphatase4 (PFKFB4), a bifunctional enzyme increasing glucose uptake and glycolytic capacity of cancer cells [53].

In contrast, recent studies suggest that HIF-1 can also act as a proapoptotic factor [54, 55]. BNIP3 is a known HIF-1 target gene that has been implicated in mitochondrial autophagy induced by hypoxia, which indicates the proapoptotic function of HIF-1 [54]. In addition, p53 is considered to be the most common inhibitor of cellular proliferation as well as inducer of apoptosis. When cells sense a decrease in oxygen availability, HIF-1 can enhance the stability of p53 and these two transcription factors cooperate with each other to induce apoptosis [55].

### 3.2. HIF-1 and Angiogenesis

Angiogenesis, the formation of new blood vessel, is essential for cancer progression. It has been shown that antiangiogenic therapy is effective in the treatment of HCC [56]. VEGF, the most potent angiogenic molecule, participates specifically in promoting vascular endothelial cell division, proliferation, and migration. Under hypoxia, HIF-1 has been proven to be a direct transcriptional activator of VEGF pathway. Currently, the multikinase inhibitor sorafenib is still the only approved drug for patients with advanced HCC and it has been demonstrated that the mechanisms that account for the antiangiogenic efficiency of sorafenib is associated with its inhibitory effect on the expression of HIF-1 and VEGF proteins, leading to a decrease in vascularization of HCC [57]. Wang et al. reported that in an experimental rat HCC model, after twenty weeks of hepatocarcinogenesis induction, the levels of HIF-1 and VEGF significantly increased, suggesting that HIF-1 and VEGF play critical roles in HCC, possibly through promoting angiogenesis [58]. Besides this, another report has also shown that acriflavine, a drug inhibiting dimerization and transcriptional activity of HIF-1, decreases the expression of VEGF, leading to potent inhibitory effects on tumor vascularization [59].

In addition to VEGF, HIF-1 also induces the expression of other angiogenic growth factors such as stromal derived factor 1 (SDF1), angiopoietin 2 (ANGPT2), placental growth factor (PGF), platelet-derived growth factor-B (PDGF-B), and stem cell factor (SCF) [60, 61]. However, further studies are warranted to further validate the effects of HIF-1 on the angiogenesis of HCC in hypoxic state to confirm the possibility of HIF-1 as a therapeutic target in HCC.

### 3.3. HIF-1 and Invasion and Metastasis

The occurrence of intrahepatic and extrahepatic tumor cell metastasis is the primary factor causing poor prognosis of patients with HCC. Invasion and metastasis consist of a series of steps and the initiation involves the acquisition of a motile phenotype by tumor cells in a process called epithelial-mesenchymal transition (EMT). Thereafter, EMT has functional requirement for loss of E-cadherin, which is a major component of the adhesion junctions that maintain epithelial integrity and polarity [62]. Several lines of evidence strongly indicate that HIF-1 may be a master regulator of EMT by upregulating transcription repressors of E-cadherin, such as Snail, Twist1, transcription factor 3 (TCF3), Zfhx1a, and Zfhx1b [63–65]. HIF-1 has been shown to promote invasion and metastasis of HCC through inducing EMT in hypoxic state. The possible associated molecular mechanism is that HIF-1 interacts with two HREs in promoter of Snail and upregulates the expression of Snail to indirectly affect levels of E-cadherin, N-cadherin, and Vimentin [66].

In addition, the degradation of the extracellular matrix (ECM) including basement membrane is another key step in tumor metastasis and this complex multistep process has been proved to be associated with matrix metalloproteinases (MMPs), especially MMP-2 and MMP-9 [67, 68]. When tumors encounter low oxygen tension, HIF-1 acts as a transcription factor upregulating the expression of MMPs in both mRNA and protein levels [69, 70]. In contrast, silencing of HIF-1 inhibits cellular metastasis through alteration of invasion-related enzymes such as MMP-2 and MMP-9, both of which are decreased with the introduction of HIF-1*α* small interfering RNA (siRNA) [71].

In patients with HCC, lymph node metastasis (LNM) as a way of tumor metastasis is closely related to a low survival rate. Xiang et al. identified 83 cancer genes that were differentially expressed in 20 pairs of HCC patients with or without LNM and found that the combination of intratumoral HIF-1*α*, VEGF, and MMP-2 may be a molecular model for predicting LNM of HCC patients [72]. Furthermore, knockdown of HIF-1*α* expression by adenovirus-mediated small hairpin RNA (shRNA) inhibits the angiogenesis and invasion in HCC cells and as a result, the levels of VEGF and MMP-2 are also repressed in endothelial cells transferred to the tumor [73]. Angiopoietin-like protein 4 (ANGPTL4), another target gene of HIF-1, promotes transendothelial migration of HCC cells in vitro and in vivo. Moreover, serum ANGPTL4 is higher in HCC patients compared with healthy control, which indicates that ANGPTL4 may be a novel prognostic marker in HCC patients [74]. In another work by Liu and coworkers, the homeobox protein PROX1 is demonstrated to be a critical factor promoting HCC metastasis, and its prometastasis activity is significantly associated with the upregulation of HIF-1*α* transcription and stabilization, which subsequently induces an EMT response in HCC cells [75]. Additionally, Zhang and coworkers also reported that *β*-catenin enhances hypoxia-induced invasion capacity in HCC cell lines by increasing the EMT-associated activity of HIF-1*α* [76].

### 3.4. HIF-1 and Resistance to Radiotherapy and Chemotherapy

Radiotherapy is an established treatment for HCC patients by killing tumor cells via induction of oxidative stress. However, radiotherapy resistance is initiated owing to producing less oxygen-free radicals in the hypoxia region of tumor. Among these mechanisms of radiotherapy resistance, the HIF-1 pathway has a profound effect on the tumor-protective response to radiotherapy via increasing the antioxidant capacity of tumors to counter the oxidative stress after irradiation [77]. The consequences of postirradiation HIF-1 activation are complex, involving both vascular protection and changing of glucose metabolism. On the one hand, upregulation of HIF-1 stimulates tumor cells to produce VEGF and other proangiogenic factors during radiotherapy, which protect the microvasculature from radiation-induced endothelial apoptosis. On the other hand, recent researches show that HIF-1 inhibition results in metabolic alterations that could enhance the therapeutic efficacy of radiotherapy [78, 79]. Based on the work by Yang et al., HIF-1 downregulation by siRNA enhances radiosensitivity in chemical hypoxic HCC cells in vitro suggesting that radiotherapy in combination with specific inhibition of HIF-1 would be expected to have a stronger anticancer effect on human hepatoma [80]. In the clinical research which evaluates the relationship between HIF-1 and responses of abdominal metastatic lymph nodes from HCC patients treated with external beam radiotherapy (EBRT), it is illustrated that HIF-1 expression in primary HCC is correlated with radiotherapy response and clinical outcome [81].

The hypoxic environment plays a critical role in promoting resistance to anticancer drugs, and the expression of HIF-1 may serve as a biomarker for better understanding of chemoresistance in cancer treatment through inducing hypoxia-elicited multiple drug resistance (MDR1) gene and increasing the expression of P-glycoprotein, a transmembrane protein associated with tumor resistance to chemotherapeutics [82]. There is recent evidence that HIF-1 is upregulated in response to doxorubicin and HIF-1-targeting strategies may enhance efficacy of doxorubicin, which is frequently used to treat many cancers including HCC [83]. Zhu et al. have elucidated the molecular mechanism by which multidrug resistance of HCC develops. Their study provided the evidence that HIF-1 controls the transcription of MDR-related genes in HepG2 cells [84]. In addition, Tung et al. found that HIF-1 acts as a major role in the acquisition of arsenic trioxide (ATO) resistance in human HCC. HIF-1-induced drug-resistance of ATO highlights the potential importance of HIF-1 as a prime molecular target to reverse ATO resistance in HCC [85].

### 3.5. HIF-1 and Prognosis

As a common malignant solid tumor, HCC is characterized by poor prognosis and treatment options are largely limited by the frequent presence of metastases. It is observed that aberrant HIF-1 activation in HCC cells is associated with the development and prognosis of HCC. It has been found that HIF-1*α* expression is detected in the sera of HCC patients at a significantly higher level than in cases of benign liver disease, suggesting that circulating HIF-1*α* level is a new biomarker for diagnosis and prognosis of HCC [86]. In the study by Simon and coworkers, HIF-1*α* was determined by quantitative RT-PCR in HCC tissue and paired nonmalignant liver tissue of 53 patients surgically treated for HCC. They concluded that the dysregulation of HIF-1*α* in apparently nonmalignant liver tissue provides a modulated environment that potentially enhances HCC recurrence after curative resection [87]. Dai et al. have investigated the expression of HIF-1 in HCC and correlated the level of HIF-1 with poor outcome. The authors found that HIF-1 in HCC plays an important role in predicting patient outcome and their study identified a potential novel mechanism contributing to prognosis of HCC [88].

In summary, higher level of HIF-1 expression might indicate a poorer prognosis in patients with HCC, and HIF-1 could be used as a novel useful biomarker for the prediction of prognosis of HCC patients. However, the conclusion is hampered by the limitations of the included studies. Further studies evaluating the significance of HIF-1 for prognosis of HCC are strongly recommended [89, 90].

## 4. HIF-1 in the Therapy of HCC

### 4.1. The Application of HIF-1 Inhibitors

Given the central role of HIF-1 in the activation of numerous pathways responsible for tumorigenesis and progression of HCC, it is not surprising that targeting HIF-1 has become a novel therapeutic strategy. Increasing small molecules have been found to inhibit HIF-1 activity through various mechanisms including decrease of mRNA transcription, downregulation of protein synthesis, and disrupting HIF-1 stabilization, inhibition of subunit heterodimerization, interference of HIF-1-DNA binding, and transcriptional activity attenuation of HIF-1 [91, 92]. Many of these agents targeting HIF-1 are already in clinical trials. For instance, YC-1(3-(5′-hydroxymethyl-2′-furyl)-1-benzyl indazole) is a potent inhibitor of HIF-1 via stimulation of FIH dependent p300 dissociation from HIF-1*α* [93]. It has been found that YC-1 effectively inhibits the migration of HCC cells through decreasing HIF-1 levels and the expression of downstream target genes, which implies that YC-1 has potential as a multipurpose anticancer drug and is worthwhile to develop for the prevention of tumor spreading [94].

In recent years, growing number of HIF-1 inhibitors are found as potential HCC therapeutic drug leads. As mentioned earlier, HIF-1 plays a significant role in HCC chemotherapy resistance and thereby limits the efficiency of sorafenib, which is the only approved targeted small molecular drug for HCC therapy. EF24, a curcumin analog, has been shown to synergistically enhance sorafenib efficacy for HCC patients through overcoming sorafenib resistance by upregulating VHL protein level and inhibiting HIF-1. This result indicates that EF24 in combination with sorafenib represents a promising strategy for the treatment of HCC [95]. A recent study shows that conjugated linoleic acid (CLA) inhibits HIF-1*α* stabilization in the HepG2 cell line and induces apoptotic cell death under hypoxic condition. This is the first report of the inhibitory effect of CLA on HIF-1*α* stabilization. Further detailed mechanisms of this compound remain to be elucidated [96]. Tanaka and his colleagues reported that LS081, with iron-facilitating activity, is capable of inhibiting Hep3B and HepG2 growth in vitro and in vivo through accelerating the degradation of HIF-1 via prolyl hydroxylases. LS081 itself did not show cytotoxic effects on cell growth in vitro. Therefore, treatment with LS081 might be a novel approach for HIF-1-targeting treatment in cancer [97]. Using New Zealand White rabbits implanted with VX2 liver tumor, Liang et al. found that HIF-1*α* protein is significantly overexpressed in transcatheter arterial embolization (TAE) treated liver tumors, and 10-hydroxycamptothecin (HCPT), a HIF-1*α* inhibitor, is demonstrated to have an inhibitory effect on HIF-1*α* expression that subsequently inhibits angiogenesis of liver tumors after TAE treatment. These results suggested that transcatheter infusion of HCPT after TAE treatment might have a better therapeutic effect against HCC [98–100].

In conclusion, several HIF-1 inhibitors have been demonstrated as potential HCC therapeutic drug. However, further investigations are needed before this strategy can be translated into clinical application.

### 4.2. HIF-1 Gene Therapy

Gene therapy, the modification of gene expression to treat diseases, has become a tremendous area of research to provide therapeutic benefits against cancers over the past 20 years. Due to the crucial role of HIF-1 for tumor growth in HCC, HIF-1 becomes a novel molecular target for gene therapy using nucleic acids, such as DNA, siRNA, shRNA and antisense oligonucleotides (ASON). These nucleic acids are delivered to target cells to modify the expression of HIF-1 and are hoped to exert anti-HCC activity by targeting HIF-1.

RNA interference (RNAi) is a gene regulation mechanism mediated by some relevant RNA molecules such as siRNA and shRNA. siRNA or shRNA is able to achieve a reliable and sequence-specific gene silencing by binding to corresponding mRNA sequences and activating a biochemical pathway leading to degradation of the mRNA, thus blocking the translation of the target mRNA into specific protein. In addition, composed of 20–24 nucleotides, microRNAs (miRNAs) also utilize the same biological machinery and regulate protein-coding gene expression, usually resulting in gene silencing [101–104]. A recent study has shown that silencing of HIF-1*α* gene using specific siRNA can significantly inhibit the proliferation of hypoxic CBRH-7919 rat hepatoma cells by inactivation of the PI3K/AKT signaling pathway. In the study, the researchers demonstrate that HIF-1*α* silencing using siRNAs could be a potential gene therapy for anticancer treatment [105]. Chen and coworkers demonstrated that RNAi against HIF-1*α* could improve the efficacy of TAE and reduce undesirable effects in the treatment of HCC by TAE through suppressing VEGF and the microvessel density (MVD). Thus, the combination of RNAi of HIF-1*α* and TAE had a marked synergistic effect on tumor growth inhibition [106].

Antisense oligonucleotide (ASO) technique is another approach widely used in gene therapy. The antisense single-strand DNA or RNA can identify the regions of mRNA and inhibit the expression of target genes. Studies have confirmed that HIF-1 antisense oligonucleotide is able to inhibit the HCC cell proliferation by reducing the gene expression and protein synthesis of HIF-1. The findings suggested that antisense technique targeting HIF-1 might be an effective gene therapy of HCC [107]. Moreover, Liu et al. have demonstrated that gene transfer of antisense HIF-1*α* downregulates the expression of HIF-1*α* as well as VEGF to inhibit tumor growth and angiogenesis, thereby enhancing the therapeutic efficacy of doxorubicin towards HCC. In summary, antisense HIF-1*α* therapy has promising utility in the treatment of HCC [108].

## 5. Conclusion and Perspectives

In conclusion, multitudinous studies have provided compelling evidence that HIF-1 plays important roles in many critical aspects of HCC tumorigenesis, progression, and metastasis. It is involved in cellular proliferation, angiogenesis, invasion, and resistance to radiotherapy and chemotherapy. Clinical data also indicate that HIF-1 overexpression is associated with poor prognosis of HCC. More importantly, HIF-1 is identified as a potential target for HCC therapy. Thus, some small-molecular inhibitors of the HIF-1 pathway have been used in combination with other therapies to improve anticancer efficacy. Despite the different approaches to identify HIF-1 pathway inhibitors that effectively inhibit liver cancer tumorigenesis, there are some limitations in the application of these agents, such as the undesirable side effects, the relatively low specificity, less obvious antitumor effects, and the lack of clinical efficacy evaluation. Thus, further studies are warranted to discover optimal therapeutic agents targeting HIF-1 for improved clinical outcomes.

HIF-1 has also been demonstrated to provide a novel target for gene therapy in HCC treatment. However, a number of questions that have yet to be answered hinder the current development of RNAi technique targeting HIF-1. Even though clinical trials are undergoing, the unassured safety of RNAi and its potential to induce unwanted immune response are issues of common concern. At the same time, effective delivery of RNA molecules is another hurdle for the RNAi-based HCC therapy. Recently, many exciting studies have improved the efficiency and selectivity of siRNA delivery utilizing viral or nonviral delivery systems mainly cationic liposomes or nanoparticles and thereby increased the confidence in the potential of HIF-1 RNAi for fighting HCC. We believe that further studies remain to be done to elucidate the important role of HIF-1 in HCC and there is still a long way to go before we can confidently promote the clinical application of HIF-1 targeting therapy for HCC.

## Figures and Tables

**Table 1:** Target genes that are transcriptionally activated by HIF-1 in cancer progression.

Effect on cancer progression	Target genes of HIF-1	References
Angiogenesis	VEGF, NOS, ET1, SDF1, ANGPT2, PDGF, leptin	[21–27]
Glucose metabolism	GLUT1, HK, LDHA, MCT4, PDK1, MXI1	[31–36]
Cell proliferation	NOS, IGF-2, TGF, C-MYC, ID2	[22, 37–40]
Cell apoptosis	p53, BNIP3, Caspase 3	[41–43]
Invasion and metastasis	MMP2, FN1, C-MET, AMF, KRT14	[44–48]
